# *Klebsiella pneumoniae* Lipopolysaccharide as a Vaccine Target and the Role of Antibodies in Protection from Disease

**DOI:** 10.3390/vaccines12101177

**Published:** 2024-10-17

**Authors:** Jernelle C. Miller, Alan S. Cross, Sharon M. Tennant, Scott M. Baliban

**Affiliations:** 1 Center for Vaccine Development and Global Health, University of Maryland School of Medicine, Baltimore, MD 21201, USA; jernelle.miller@som.umaryland.edu (J.C.M.); across@som.umaryland.edu (A.S.C.); stennant@som.umaryland.edu (S.M.T.); 2 Department of Medicine, University of Maryland School of Medicine, Baltimore, MD 21201, USA

**Keywords:** *Klebsiella pneumoniae*, O-antigen, O-polysaccharide, lipopolysaccharide, capsule masking, antibody, protection, vaccine

## Abstract

*Klebsiella pneumoniae* is well recognized as a serious cause of infection in healthcare-associated settings and immunocompromised individuals; however, accumulating evidence from resource-limited nations documents an alarming rise in community-acquired *K*. *pneumoniae* infections, manifesting as bacteremia and pneumonia as well as neonatal sepsis. The emergence of hypervirulent and antibiotic-resistant *K*. *pneumoniae* strains threatens treatment options for clinicians. Effective vaccination strategies could represent a viable alternative that would both preempt the need for antibiotics to treat *K*. *pneumoniae* infections and reduce the burden of *K*. *pneumoniae* disease globally. There are currently no approved *K. pneumoniae* vaccines. We review the evidence for *K*. *pneumoniae* lipopolysaccharide (LPS) as a vaccine and immunotherapeutic target and discuss the role of antibodies specific for the core or O-antigen determinants within LPS in protection against *Klebsiella* spp. disease. We expand on the known role of the *Klebsiella* spp. capsule and O-antigen modifications in antibody surface accessibility to LPS as well as the in vitro and in vivo effector functions reported for LPS-specific antibodies. We summarize key hypotheses stemming from these studies, review the role of humoral immunity against *K*. *pneumoniae* O-antigen for protection, and identify areas requiring further research.

## 1. Introduction

### 1.1. Clinical and Epidemiological Data from a Global Perspective

#### 1.1.1. Infections Caused by *Klebsiella pneumoniae*

*Klebsiella pneumoniae* is a Gram-negative, encapsulated member of the family *Enterobacteriaceae* [1]. It typically causes pneumonia and sepsis, particularly in hospitalized individuals as well as those who are immunosuppressed. *K*. *pneumoniae* can also give rise to meningitis and urinary tract infections, and infections with hypervirulent clones are associated with invasive disease, including liver and lung abscesses, in otherwise healthy adults [2,3].

#### 1.1.2. Hospital-Acquired Infections

*Klebsiella* spp. have been identified amongst the three most common causes of healthcare-associated infections (HAIs) in the U.S. [4,5,6] and Europe [7]. HAIs include surgical site infections, ventilator-associated pneumonia, catheter-associated urinary tract infections, central line-associated bloodstream infections, device-associated HAIs and other bloodstream infections. *K. pneumoniae* infections are a major public health concern for residents in long-term acute care facilities [6,8,9,10]. A major risk factor for infection with *K. pneumoniae* is prior colonization with the bacterium [11,12,13,14].

#### 1.1.3. Community-Acquired Infections

Although *K. pneumoniae* is commonly acquired in hospitals or associated with healthcare settings, it can also be acquired in the community [15] and has been associated with pulmonary infections in alcoholics since the organism was first described by Carl Friedlander in 1882 [16]. Community-acquired pneumonia and bacteremia are relatively rare in high-income countries but are more common in Asia and Africa [3,17,18]. Interestingly, an association between bacteremic *K. pneumoniae* pneumonia and alcoholism was observed in South Africa and Taiwan [3]. In the mid-1980s in Taiwan, reports of *K. pneumoniae* infections associated with unusual disease manifestations (e.g., liver abscesses and other invasive syndromes) began to appear, which was distinct from the “classical” *K. pneumoniae* strains that were more commonly associated with HAIs. In addition to causing invasive clinical manifestations, hypervirulent clones of *K. pneumoniae* frequently produce a hypermucoviscous phenotype on culture media evidenced via a positive string test [19,20]. These strains may also express genetic determinants associated with virulence (e.g., siderophores or transcriptional regulators of capsule production and export) and display enhanced virulence in murine respiratory disease models [21]. Hypervirulent and hypermucoviscous variants of *K. pneumoniae* that cause community-acquired infections are also circulating in the U.S. [22,23] and elsewhere in the world [24,25,26].

#### 1.1.4. Neonatal Sepsis

There is growing recognition that *K. pneumoniae* is a major cause of neonatal sepsis in resource-poor settings. A recent meta-analysis of studies published between 1980 and 2018 showed that *K. pneumoniae* was responsible for 21% of culture-positive bacteremia or sepsis cases in 84,534 neonates from 26 countries in sub-Saharan Africa [27]. *K. pneumoniae* was the most common cause of neonatal sepsis in the Burden of Antibiotic Resistance in Neonates from Developing Societies (BARNARDS) study [28,29,30]. BARNARDS was a prospective observational cohort study conducted in 12 sites in Bangladesh, Ethiopia, India, Pakistan, Nigeria, Rwanda, and South Africa to determine the incidence of neonatal sepsis as well as pathogens and factors associated with neonatal sepsis. Interestingly, 56.9% of *K. pneumoniae*-associated sepsis cases (*n* = 123) were delivered via caesarean section, suggesting that a significant proportion of neonates do not acquire their *Klebsiella* spp. infection by traversing the birth canal. The importance of *K. pneumoniae* in neonatal sepsis was also demonstrated by the Child Health and Mortality Prevention Surveillance (CHAMPS) study. Out of 274 child deaths due to infectious causes in Mozambique, South Africa, Kenya, Mali, and Bangladesh, *K. pneumoniae* was the most common pathogen that contributed to mortality (31%) [31].

#### 1.1.5. Antimicrobial Resistance

*K. pneumoniae* was recognized as a pathogen of importance in 2008 when the term ESKAPE (*Enterococcus faecium*, *Staphylococcus aureus*, *K. pneumoniae*, *Acinetobacter baumannii*, *Pseudomonas aeruginosa*, and *Enterobacter* spp.) was first coined [32]. These bacteria have developed resistance to several (>3) classes of antibiotics and finding new therapeutics or preventives will be important to control these infections. The U.S. Centers for Disease Control and Prevention released an Antibiotic Resistance Threats report in 2013 and an update in 2019. These reports categorized pathogens based on clinical impact, economic impact, incidence, 10-year projection of incidence, transmissibility, availability of effective antibiotics, and barriers to prevention. Pathogens were categorized as urgent, serious, or concerning threats. Carbapenem-resistant *Enterobacteriaceae* were classified as an urgent threat and extended-spectrum beta-lactamase-producing *Enterobacteriaceae* as a serious threat. Carbapenems have been considered as a “last resort” treatment for multidrug-resistant *K. pneumoniae* [33,34], and carbapenem-resistant *K. pneumoniae* (CRKP) are now ranked highest on the Bacterial Priority Pathogens List for 2024 published by the World Health Organization (WHO) [35]. Multiple studies report concerningly high levels of antibiotic resistance amongst *Enterobacteriaceae* but particularly resistance of *Klebsiella* spp. and *Acinetobacter* spp. to carbapenems [6,7,34,36,37,38]. Limited therapeutic options for infection with multidrug resistant *K. pneumoniae* results in increased mortality, longer hospital stays, and increased healthcare costs [39]. A recent study showed that there were ~4.95 million deaths associated with bacterial antimicrobial resistance (AMR) globally in 2019 which included ~1.27 million deaths directly attributable to bacterial AMR [40]. *K. pneumoniae* was responsible for 17.5% of the deaths associated with AMR and 19.9% of the deaths attributable to AMR.

### 1.2. Vaccination to Combat K. pneumoniae Infections

#### 1.2.1. Initial Vaccine Development Efforts Targeting the Capsular Polysaccharide

There are currently no FDA-licensed vaccines to prevent *K. pneumoniae* infections and associated mortality. The first *K*. *pneumoniae* vaccine candidates to undergo clinical evaluation targeted the capsular polysaccharide (CPS, K-antigen), a well-established virulence factor which coats the bacterial surface in high abundance. The *K. pneumoniae* capsule plays an important role in protecting against host immune defenses, and the most well-studied immune evasion mechanisms include inhibiting opsonophagocytosis, suppressing bacterial lysis by complement and antimicrobial molecules (e.g., lactoferrin), and blocking innate immune activation. We refer the reader to other reviews for further information [1,41].

This choice to target the capsule for these early vaccines was influenced in part by previous experience with the multivalent pneumococcal CPS-based vaccines that were in development at the time and that some of the anti-pneumococcal CPS antibodies displayed cross-reactivity to *K. pneumoniae* CPS. At least 77 serologically distinct K types are known to exist for *Klebsiella* spp., while 147 different K loci have been reported via genotyping [42]. Based on the seroepidemiology of the *K. pneumoniae* CPS in human disease, 24 K-types were chosen to prepare a vaccine that would cover ~80% of clinically relevant strains. After strong preclinical data, this vaccine underwent multiple clinical studies in the 1980s and 1990s resulting in the production of a hyperimmune immunoglobulin for intravenous use (H-IVIG) [43,44,45,46]. A single infusion of this H-IVIG was administered to patients entering the intensive care unit of Veterans Affairs (VA) medical centers to determine if it would prevent the onset of *K. pneumoniae* infection. While there was some suggestion of a reduction in bacteremia during the first week of follow-up, by the end of the observation period there was no significant effect, probably due to the waning of circulating antibody levels [47]. The waning of antibody levels after passive administration for prophylaxis emphasized that active immunization might be a more effective preventive strategy. Subsequently, there was relatively little interest in developing *K*. *pneumoniae* vaccines.

#### 1.2.2. Renewed Interest in Vaccine Development and Major Target Populations

The recent dramatic increase in *K. pneumoniae* infections that are resistant to “last resort antibiotics” and their emergence as a leading pathogen in neonatal sepsis, particularly in low- and medium-income countries, has renewed efforts aimed at preventing these infections. An effective vaccine represents a cost-effect measure to mitigate disease burden, thereby reducing the need for antimicrobial therapy and may decrease the transmission of these pathogens. Given the importance of *K. pneumoniae* in the hospital setting, a major target population for a *K. pneumoniae* vaccine are individuals who will be entering hospitals for surgical procedures, admitted to a ward, or who will be entering long-term acute care facilities. Another major group that *K. pneumoniae* vaccines should target are neonates. Here, maternal immunization with a *K. pneumoniae* vaccine during pregnancy would protect neonates from sepsis due to *K. pneumoniae*, similar to the approach that is being taken for vaccines that protect against Group B streptococcus in newborns [48]. Finally, given the growing antibiotic resistance of *K. pneumoniae*, vaccines could also be used to reduce antibiotic-resistant *K. pneumoniae* infections in the community [49].

## 2. LPS-Based Vaccination Strategies Against *K*. *pneumoniae*—Where Do We Stand?

As an alternative to the CPS for *K. pneumoniae* vaccine development, lipopolysaccharide (LPS) is an attractive vaccine antigen given its abundant expression in the bacterial outer membrane and its central role in *Klebsiella* spp. virulence. A complete LPS molecule consists of three structural domains: lipid A (endotoxin), a conserved core oligosaccharide, and the O-polysaccharide (OPS, O-antigen), which is a repeating glycan polymer that is antigenically diverse across serotypes. LPS is the primary means by which *K. pneumoniae* evades complement-mediated killing [50,51,52,53,54,55,56]. Strains possessing longer LPS (the length of which is conferred by the number of O-antigen repeating units) or O-antigen branching modifications demonstrate greater serum resistance likely by blocking membrane attack complex formation or sequestering it at an ineffective distance from the bacterial surface [52,56]. Expression of O-antigen enables *K. pneumoniae* to invade systemically following pulmonary colonization in murine pneumonia models [57], and certain *K. pneumoniae* O-antigens induce poor pro-inflammatory immune responses from human monocytes and neutrophils [58,59]. We refer the reader to other reviews for further information [1,41].

LPS has received comparatively less attention for *K. pneumoniae* vaccine development than CPS as it was unclear whether this polysaccharide antigen would be surface exposed (and thus recognizable by protective antibodies) in the presence of the capsule and under physiological conditions. In the late 90’s and early 00’s, a major shift in the field occurred when two independent groups developed highly specific anti-OPS antisera to type 1,016 clinical isolates of *K. pneumoniae* from the US, Denmark, Germany, and Spain, revealing the existence of nine unique O-antigen serogroups. Remarkably, only four (namely O1, O2, O3, and O5) accounted for the bulk (>79%) of clinical infections [60,61]. A high prevalence of the same four O-types was also documented in a collection of 645 *K. pneumoniae* bloodstream isolates from 13 countries throughout 2005 to 2017 [38]. As a result of these studies and others demonstrating accessibility of subcapsular targets to antibodies (discussed below), interest in targeting LPS, and in particular the O-antigen, for *K. pneumoniae* vaccine development has reignited, considering that a 4-valent OPS-based vaccine with broad global coverage would likely be more commercially viable than a minimally 24-valent CPS-based vaccine.

### 2.1. Clinical Evaluation of K. pneumoniae Vaccines

#### 2.1.1. O-polysaccharide Bioconjugates

As of this review, the only OPS-based *K. pneumoniae* vaccine candidate to have entered clinical development is a quadrivalent bioconjugate vaccine (Kleb4V) targeting serotypes O1, O2 (including the O2a and O2afg subtypes), and O3b, which is being developed by GSK (London, U.K.) and LimmaTech Biologics AG (Schlieren, Switzerland). A Phase I/II study to assess the safety and immunogenicity of Kleb4V was completed in 2022 (NCT04959344).

#### 2.1.2. Whole Cell Vaccines

*Klebsiella* spp. have been included as part of various polymicrobial preparations or their cellular lysates that are described as both immunomodulators and vaccines. Growing clinical evidence suggests that oral or sublingual administration of mixed respiratory bacterial lysates is effective in reducing the frequency, duration, and severity of respiratory tract infections (RTIs) as well as reducing antibiotic usage [62]. Some examples of commercial bacterial lysates available for treating RTIs include Broncho-Vaxom^®^ (OM-85 BV) and Ismigen^®^, which are oral capsules of killed *K. pneumoniae*, *Klebsiella ozoneae*, *Streptococcus pyogenes*, *Streptococcus pneumoniae*, *Streptococcus viridans*, *S. aureus*, *Moraxella catarrhalis*, and *Haemophilus influenzae*; Lantigen B^®^, an oral suspension of inactivated *K. pneumoniae*, *S. pyogenes*, *S*. *pneumoniae*, *S*. *aureus*, *M. catarrhalis*, and *H. influenzae*; and Luivac^®^ (LW-50020), an oral tablet which contains similar respiratory pathogens as LantigenB^®^ but also includes lysate from *Streptococcus mitis*.

Polymicrobial formulations containing *K. pneumoniae* have also demonstrated clinical benefit against recurrent urinary tract infections [63,64]. These include Uromune^®^ (MV-140), a sublingual spray composed of heat-inactivated *K. pneumoniae*, *Escherichia coli*, *Proteus vulgaris*, and *Enterococcus faecalis*; Solco-Urovac^®^, a vaginal suppository/intramuscular injection consisting of heat-killed *K. pneumoniae*, *E. coli*, *Proteus mirabilis*, *E. faecalis,* and *Morganella morganii*; and Urostim^®^, an oral tablet containing bacterial lysates of *K. pneumoniae*, *E. coli*, *P. mirabilis*, and *E. faecalis*.

Another mixed bacterial suspension is Dentavax^®^, which is composed of killed *K. pneumoniae*, *S. aureus*, *S. pyogenes*, *Lactobacillus acidophillus*, and *Candida albicans*. Dentavax was shown to elicit systemic and mucosal T and B cell responses after oral delivery in human volunteers [65]; however, it has not yet been evaluated for efficacy in humans.

### 2.2. K. pneumoniae Vaccines in Preclinical Development

A plethora of *K. pneumoniae* vaccine candidates where LPS is either specifically targeted or would be expected to play a significant role in the vaccine-induced immune response have been described. In Table 1, we summarize each of these candidates, providing information on administration routes, vaccine serotype(s) as well as any immunogenicity and efficacy findings that were reported.

#### 2.2.1. Whole Cell Vaccines and Outer Membrane Vesicles

The protective potential of inactivated *K. pneumoniae* was first reported in 1970 where mice that were immunized with formalin-treated bacteria survived a lethal parenteral challenge with the homologous organism [71]. Since then, *K*. *pneumoniae* vaccines consisting of either heat-killed, encapsulated bacteria [72,73] or outer membrane vesicles (OMVs) [92,93] have been described. Furthermore, attenuating *K*. *pneumoniae* to produce vaccine strains has been pursued through various methods including disruption of iron uptake systems (via *tonB* deletion) [74] and imposing glutamate auxotrophy (via *murI* deletion) [75], as well as inhibiting capsular polysaccharide production or outer membrane protein biosynthesis (via *magA* or *kbvR* deletion) [76,77]. These studies collectively demonstrated that whole-cell- and OMV-based *K*. *pneumoniae* vaccines are immunogenic and protective in murine models of systemic or pulmonary challenge.

A link between protection elicited by *K. pneumoniae* whole-cell vaccines and antibodies recognizing subcapsular antigens was explored by Hsieh et al. using a capsule-deficient (Δ*magA*) mutant of *K*. *pneumoniae* (serotype O1) [77]. Mice immunized with this strain were protected from lethal challenge with the encapsulated parent strain (O1:K2), and passive transfer of post-vaccination antisera led to a reduction in tissue bacterial burden compared to control animals.

#### 2.2.2. Lipopolysaccharide

Purified *K*. *pneumoniae* LPS can be utilized as a vaccine antigen without sacrificing the robust protection observed with whole bacteria. As a proof of principal, Tomás et al. immunized mice with O1 LPS and then challenged them intraperitoneally (IP) with various strains of *K*. *pneumoniae* expressing the same O-type (O1) but different K-types (K1, K2, K21, and K62) [79]. LPS-vaccinated mice were significantly protected against serotype K2 challenge, whereas they were much more susceptible to an O1:K1 strain. When challenge occurred with the K21 and K62 serotypes, which were less virulent in this study, moderate protection was achieved in vaccinated mice. The protective potential of LPS from *Klebsiella* spp. was verified in another study where O1 LPS immunization in mice afforded protection against an O1:K2 strain of *K. pneumoniae* delivered intravenously [67]. Immunization with LPS from *K. pneumoniae* was also effective in a mouse urinary tract infection model where the number of viable bacteria in the kidneys was diminished in O1 LPS-vaccinated mice after intraurethral challenge with O1:K2 *K. pneumoniae* [68]. Collectively, these studies demonstrated the efficacy of LPS immunization against experimental *K*. *pneumoniae* infection, although the degree of protection varied considerably depending on the virulence and K-type of the challenge strain.

To enhance its immunogenicity, LPS from *Klebsiella* spp. was incorporated into multilamellar vesicles consisting of phosphatidylcholine, phosphatidylserine, and cholesterol [69]. The LPS–liposome formulation was immunogenic in rats, eliciting elevated specific B cell responses within the spleen compared to free LPS, and immunized animals were protected against lobar pneumonia induced by an O1:K2 strain. In another study*, K. pneumoniae* LPS was displayed on sodium alginate microparticles and used to immunize mice via the intranasal (IN), intramuscular (IM), or intratracheal (IT) routes. [70]. All three routes were immunogenic and protective against IT challenge, although serum IgG responses as well as lung bacterial burden were highest in IM-immunized mice. Compared with controls, vaccinated mice failed to develop pulmonary edema following IT infection with a *K. pneumoniae* O1:K2 strain. Interestingly, higher cellular infiltrate and alveolar septum swelling were evident within the lungs of mice immunized with the microparticle formulations compared to those immunized with free LPS, presumably due to the adjuvanticity of the microparticles.

#### 2.2.3. Glycoconjugate Vaccines

Lipid A is a potent immune stimulator and limits the prospects of LPS for vaccine development due to safety concerns. However, mild acid treatment removes the toxic lipid A moiety of LPS leaving behind the core and O-polysaccharide (COPS) domains. In the absence of lipid A, plain bacterial polysaccharide antigens tend to behave as T-independent type II antigens, thus promoting weak humoral immunity characterized by a lack of class switching, affinity maturation, and long-term B cell memory [94]. Coupling to a T-dependent protein carrier overcomes this limitation by recruiting T cell help and has been explored as a platform for candidate *Klebsiella* vaccines.

##### Conventional Core and O-polysaccharide Conjugates

The proof-of-concept for *Klebsiella* glycoconjugate vaccines was demonstrated by Chibber et al. where COPS from an O1 strain was chemically conjugated to tetanus toxoid (TT) [78]. Immunization with the COPS:TT conjugates produced an LPS-specific serum antibody response in rats that was associated with protection against pulmonary challenge with an O1:K2 strain of *K. pneumoniae*.

Hegerle et al. described a quadrivalent glycoconjugate vaccine targeting *K. pneumoniae* serotypes O1, O2, O3, and O5 [79]. In this study, the COPS molecules were chemically coupled to *P*. *aeruginosa* flagellin A (FlaA) or flagellin B (FlaB) proteins produced recombinantly in *E. coli*. Active immunization of mice with either the O1 COPS:FlaA or O1 COPS:FlaB conjugates demonstrated a consecutive rise in serum anti-COPS IgG after each of three vaccine doses. An equal mixture of the four conjugates was used to immunize rabbits, which revealed no evidence of antigen interference as measured with serum anti-COPS IgG titers. Furthermore, passive delivery of post-vaccination rabbit sera to naïve mice demonstrated reduced bacterial burden in various tissues following intravenous infection with *K. pneumoniae* O1 and O3 strains. Mice were also protected against lethal intravenous challenge with an O1:K2 strain.

More recently, a bivalent *Klebsiella*/*Pseudomonas* glycoconjugate vaccine was described wherein *K. pneumoniae* and *P. aeruginosa* COPS were conjugated to bovine serum albumin (BSA) [80]. Mice were immunized with either the monovalent COPS:BSA conjugates or a combination of the two. All mice subjected to vaccination, regardless of formulation, were significantly protected against *K. pneumoniae* challenge compared to the controls.

##### O-polysaccharide Bioconjugates

Bioconjugation is the process where recombinant bacteria are engineered to express an antigenic polysaccharide, a protein carrier, and an oligotransferase (OTase) that catalyzes the transfer of a glycan of interest to the carrier [95]. An O2 O-antigen-based bioconjugate vaccine was described where a clinical isolate of *K. pneumoniae* was engineered to express a cholera toxin B subunit (CTB) along with the OTase PglL [83]. The O-antigen ligase *waaL* was deleted from this strain to inhibit LPS biosynthesis and enable the manufacture of a vaccine containing O2 OPS covalently linked to CTB. Immunization of mice with purified O2:CTB bioconjugates elicited serum anti-LPS IgG of multiple subclasses and protected against systemic challenge with the parent O2 strain.

By contrast, Peng et al. found that O2 O-antigen based bioconjugates were weak immunogens in mice [84]. To improve their immunogenicity and efficacy, this group developed a nanoconjugate vaccine that uses self-assembling CTB as the carrier protein. This nanoparticle-based carrier system was inserted into an *E*. *coli* strain lacking its own LPS biosynthesis machinery (Δ*waaL*, Δ*waaH-L*) and expressing both PglL and the *K. pneumoniae* O2 OPS gene cluster. The resulting O2 OPS:CTB nanoconjugates were shown to elicit superior serum LPS-specific antibody responses and protection against both systemic and pulmonary challenges compared to traditional, non-self-assembling bioconjugates. Protection with nanoconjugate vaccination was associated with clearance of bacteria across multiple tissues and blood. The same group explored a self-assembling AP205 bacteriophage coat protein as an alternative nanoconjugate carrier where either O1 or O2 O-antigens were in vivo coupled to the virus-like particle using SpyCatcher/SpyTag chemistry [85]. The strain of *E. coli* described above was further engineered to reduce unwanted O-antigen side chain modification (Δ*yfdGHI*) and to limit endotoxin contamination (Δ*lpxM*). The resulting O1 and O2 OPS:AP205 nanoconjugates were found to be immunogenic in mice and protective against systemic challenge with the homologous *K. pneumoniae*. Robust protection was achieved with up to 1/4th the initial vaccine dose for O1 OPS:AP205, whereas doubling the dose of the O2 OPS:AP205 vaccine was needed to increase the degree of protection.

Lastly, Wantuch et al. described an O1 bioconjugate vaccine where the genetically detoxified recombinant exotoxin A from *P*. *aeruginosa* (EPA) was used as the carrier protein (constructed in an *E. coli* Δ*waaL* strain expressing a PglS OTase) [86]. Immunization of mice with the O1 OPS:EPA bioconjugate generated serum IgG that bound to whole bacteria via ELISA; however, unlike other reports of *K. pneumoniae* bioconjugates, vaccinated mice were not protected against lethal pulmonary or bacteremic challenge with O1:K2 *K*. *pneumoniae*. This group has also reported on a heptavalent bioconjugate formulation that targets the four main *K. pneumoniae* O serotypes and their subtypes (O1 (including the O1a and ᴅ-galactan-III + subtypes), O2 (including the O2a and ᴅ-galactan-III + subtypes), O3b, and O5) [87]. The bioconjugates were produced in PglS OTase-expressing strains of *E. coli* Δ*waaL* or *K. pneumoniae* Δ*waaL*Δ*wcaJ*, which lacks the ability to synthesize its own capsule. Monovalent and heptavalent O-antigen bioconjugates were immunogenic in mice, producing serum IgG that reacted with the purified conjugates (via Western blot) and the unencapsulated vaccine reagent strains (via whole-cell ELISA). Binding of serum IgG generated by heptavalent immunization to a panel of *K. pneumoniae* isolates was either equivalent to monovalent antisera or in some cases better, depending on the strain. Protection experiments to assess vaccine efficacy were not performed in this study.

##### Semi-Synthetic O-polysaccharide Conjugates

Oligosaccharides pertaining to the OPS repeating units from *K. pneumoniae* serotypes O1, O2 (including the O2aeh O2ac, and ᴅ-galactan-III + subtypes), O3, and O5 were synthesized separately and each conjugated to CRM_197_ [81,82]. Conjugate-immunized mice mounted serum IgG responses against the homologous synthetic glycans, and vaccine-induced O1-, O2-, and O5-specific IgG was shown to recognize native LPS. The immunogenicity of the O1, O2, and O5 CRM_197_-conjugates was also confirmed in rabbits. Preclinical protection data have not yet been shared. These semi-synthetic vaccines were developed by Vaxxilon AG (now Idorsia Pharmaceuticals Ltd., Allschwil, Switzerland).

#### 2.2.4. Multiple Antigen-Presenting System

Polysaccharide and protein antigens can be integrated into a single complex using the multiple antigen-presenting system (MAPS), which is being developed by GSKAffinivax Inc. (Boston, MA, U.S.) [96]. Here, a backbone polysaccharide is biotinylated and linked to immunogenic proteins fused to rhizavidin. OPS are chemically linked to this backbone polysaccharide. This approach was recently applied to create a 12-valent MAPS vaccine against *K. pneumoniae* and *P. aeruginosa*. Four *K. pneumoniae* COPS (O1, O2, O3, and O5) and eight *P. aeruginosa* COPS antigens were employed [88]. Pathogen-relevant carrier proteins were also utilized, including MrkA, the major subunit of *K. pneumoniae* type 3 fimbriae. This vaccine elicited strong antibody responses in mice and rabbits. Mouse antisera was shown to bind to *K. pneumoniae* of varying capsule types. Passive transfer of rabbit antisera to naïve mice protected them from lethal IP challenge with O1:K2 *K. pneumoniae*. Protection was associated with clearance of bacteria from the bloodstream and spleen. COPS-specific antibodies were shown to promote opsonophagocytosis, whereas antibodies recognizing MrkA blocked adherence of the bacteria to epithelial monolayers.

#### 2.2.5. Core Oligosaccharide

The core oligosaccharide component of LPS is situated proximal to the bacterial membrane. The inner domain of the core is highly conserved across Gram-negative bacteria, and several groups have described active and passive vaccination strategies where this domain is targeted [97]. Cross et al. utilized a rough (Rc) mutant of *E. coli* (strain J5), which expresses truncated LPS lacking both the OPS and outer core regions, to purify the lipid A-inner core as a vaccine antigen. The J5 inner core lipooligosaccharide was delipidated to reduce endotoxicity and then noncovalently complexed with an outer membrane protein (OMP) from group B *Neisseria meningitidis*. Immunization of mice and rats with the core-OMP complexes elicited robust anti-core IgG in both serum and bronchoalveolar lavage fluid and supported protection against O1:K2 *K. pneumoniae* across multiple lethal infection models including oral challenge of neutropenic rats, subeschar challenge of flame-burned mice, and intranasal challenge of mice. [89,90,91].

## 3. Humoral Immunity to *K. pneumoniae* LPS and Protection Against Infections

### 3.1. Passive Transfer Highlights a Protective Role for Anti-LPS Antibodies

Early passive immunization studies in the 1960s and 1970s found that mice treated with antisera derived from rough mutants of *Salmonella enterica* serovar Typhimurium or *E*. *coli* were protected against fatal *Klebsiella* spp. bacteremia, highlighting the protective nature of antibodies recognizing either the conserved core oligosaccharide of LPS or other subcapsular antigens [98,99]. With the advent of monoclonal antibody (mAb) technology, other groups identified anti-lipid A/core mAbs that upon passive transfer to mice provided a survival benefit against endotoxemia as well as bacteremia caused by *K. pneumoniae* strains expressing K2 or K3 capsules [100,101]. In the late 1990s, Rukavina et al. were the first to describe a mouse mAb recognizing the *K*. *pneumoniae* O1 O-antigen that was protective against experimental sepsis induced by an O1:K2 strain [102]. Antibody pre-treated mice responded with reduced bacterial burden across various organs relative to controls that was accompanied by declines in lung cellular infiltrate.

Twenty years later, protective *K. pneumoniae* LPS-specific antibodies were identified from humans. Using peripheral blood and tonsils from either healthy donors or convalescent patients as a source of memory B cells, Pennini et al. isolated two serotype-specific mAbs (anti-O1 and anti-O2 IgG1) [33]. Both mAbs were strongly protective against experimental pneumonia (when administered therapeutically) and bacteremia (when administered prophylactically) in mice, and their efficacy against pneumonia was enhanced in the presence of antibiotics. The anti-O1 mAb also displayed therapeutic activity in a murine burn-wound infection model, significantly reducing deep tissue colonization (lung, liver, and spleen) without impacting bacterial burden at the site of infection within the skin. There is only one description of an anti-O1 IgG3 mAb of murine origin that was not protective against experimental infection with an O1:K2 strain, which the authors attributed to the weak affinity of this clone [101].

Humoral immunity to *K. pneumoniae* LPS and capsular polysaccharide (CPS) was characterized in a study of 33 hospitalized patients with blood culture-confirmed CRKP infections [103]. Anti-LPS and anti-CPS antibodies were found to varying degrees in the plasma from these patients. Purified total IgG effectively opsonized the patient-derived isolates, displaying serum bactericidal and opsonophagocytic activity in vitro and decreasing bacterial virulence in a murine pulmonary infection model. Interestingly, when anti-CPS antibody was depleted, the remaining IgG antibodies were ineffective at clearing bacteria from the lungs after pulmonary challenge. Depletion of CPS-specific IgG partially inhibited opsonophagocytosis of *K. pneumoniae* strains expressing the homologous K-antigens (40–59% reduction compared to non-depleted IgG).

### 3.2. Effector Functions of O-Antigen-Specific Antibodies

**Figure 1:**
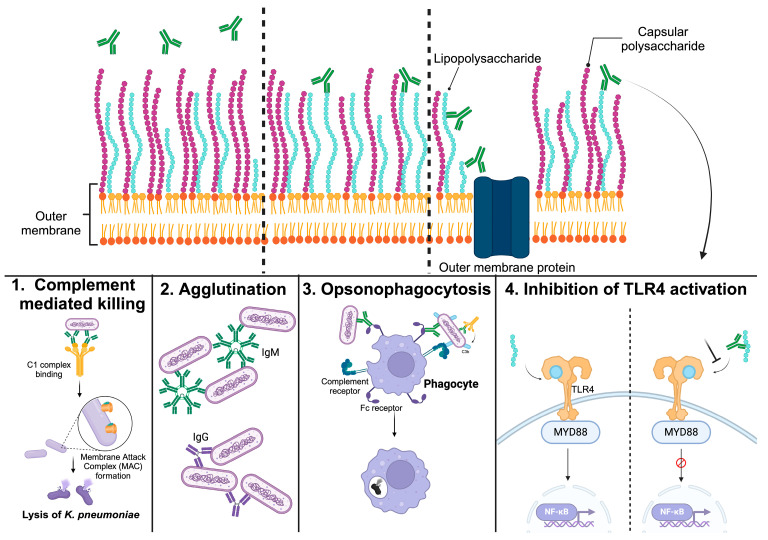
Potential interactions of LPS-specific antibodies with encapsulated *K*. *pneumoniae* and prospective effector functions for protective anti-LPS antibodies. Created in BioRender. Permala Booth, J. (2023) BioRender.com/l04l974. (**Top** panels) The configuration of capsular polysaccharide (CPS) and lipopolysaccharide (LPS) in the outer membrane of *Klebsiella* varies in confluency and length, allowing for the binding of anti-LPS antibodies. Very long CPS chains may conceal LPS from antibody recognition or even hamper the effector function of bound antibodies (**top left**). However, LPS could theoretically become accessible to antibodies when CPS and O-antigen chains are comparable in length (**top middle**) or if channels in the extracellular polysaccharide layer are sufficiently sized to accommodate antibody binding to subcapsular targets (**top right**). (**Bottom** panels) Once LPS-specific antibodies are bound, they may elicit various effector functions to control *K. pneumoniae* infection. (1) Complement-mediated killing—assembly of complement proteins and formation of the membrane attack complex leads to pore formation on the outer membrane, resulting in bacterial lysis. (2) Agglutination—antibodies can bind multiple bacteria at once to produce large immune complexes which can lead to bacterial growth inhibition, immune cell recognition, and death; (3) Opsonophagocytosis—antibody–antigen complexes and complement factors can mediate uptake by phagocytic cells via complement or Fc receptors resulting in bacterial lysis. (4) Inhibition of TLR4 activation—LPS is recognized by the Toll-like receptor 4 (TLR4)–MD2 complex, which leads to the recruitment of adaptor molecules such as MyD88, activation of the transcription factor NF-kB, and the production of inflammatory cytokines. An overexuberant immune response can lead to septic shock in the host. By blocking LPS recognition by the TLR4–MD2 complex, anti-LPS antibodies can inhibit the TLR4 signaling cascade and reduce the toxicity of LPS.

Antibodies specific to surface polysaccharides can overcome *K. pneumoniae* resistance mechanisms to phagocytosis and complement-mediated killing, where the constant region (Fc) of bound antibodies engages either the classical complement pathway or Fc receptors on phagocytes. Investigations into the effector functions of antibodies targeting *Klebsiella* spp. O-antigens often report these molecules as mediating opsonophagocytic activity (OPA). Other anti-bacterial mechanisms, however, have been documented, including agglutination, serum bactericidal activity (SBA), and TLR4-MD2 signaling inhibition (Figure 1).

Investigations into the in vivo mechanisms of action for anti-O antibodies are beginning to emerge. An O2-specific IgG1 mAb, which blocked TLR4 activation and promoted SBA and OPA in vitro, required neither Fc-mediated functions nor active complement to protect against systemic challenge with a CRKP clinical isolate belonging to sequence type (ST) 258, highlighting the importance of antibody Fab properties for protection [55,104]. Cohen et al. described two human anti-O-antigen IgG1 mAbs that exhibited similar opsonophagocytic and complement-mediated killing activity in vitro [105]. Passive immunization experiments revealed that delivery of one mAb prior to challenge was effective against lethal pneumonia (but not endotoxemia) while the other mAb displayed an opposite pattern of activity. Interestingly, both mAbs were protective against pneumonia and endotoxemia when given therapeutically. In the setting of prophylaxis, antibody-mediated protection against pneumonia was multi-faceted, requiring neutrophils, Fc-mediated mechanisms, TLR4 complex signaling, and IL-17 production. Protection was also associated with an increase in γδT cells.

### 3.3. Surface Accessibility of Core and O-Antigen in Encapsulated K. pneumoniae

#### 3.3.1. Initial Binding Studies with O1 Antisera and O1 Strains

The O-antigen was first proposed as an integral component of *Klebsiella* spp. serology in 1949 by Fritz Kauffmann [106]. An important technical issue was noted in that rabbit antibodies with specificity for subcapsular antigens, including the O-antigen, were incapable of agglutinating *Klebsiella* spp. strains expressing mature capsules. Unencapsulated cultures, where the O-antigen is completely exposed at the cell surface, were therefore recommended for O-typing *Klebsiella* spp. The notion that antibodies could interact with subcapsular epitopes and mediate an antibacterial effect remained controversial for some time.

Over three decades later, evidence that O-specific antibodies could penetrate the *Klebsiella* spp. capsule layer came when Williams et al. compared the opsonic activity of antisera from rabbits immunized with either serotype O1 strains (bearing K1 or K2 capsules) or isogenic, acapsular mutants derived from nitrosoguanidine mutagenesis [51,107]. In these studies, immunization with O1:K1 or O1:K2 *K*. *pneumoniae* yielded antibodies that readily bound to their homologous encapsulated strains, promoting uptake into human neutrophils and increasing hydrophobicity at the bacterial surface. By contrast, antibodies raised against capsule-deficient bacteria were only effective at opsonizing the O1:K2 strain, suggesting that O1 LPS was exposed to antibodies in the presence of K2 but not K1 CPSs. Interestingly, immunoadsorption experiments with whole cells found that both K1 and K2 serotypes can adsorb anti-O1 antibodies to some degree [107], suggesting that the K1 antigen can potentially mask the presence of bound anti-O antibodies. The reduced surface exposure of O-antigen was found not to be unique to serotype K1. Tomás et al. tested whether O-specific antisera from rabbits could bind to a panel of O1 *K. pneumoniae* strains from 22 different K types. This work expanded the list of impermeable capsules to include K1, K10, and K16 [66].

Confirmation that anti-O1 antibodies could efficiently penetrate K2 but not K1 capsules was obtained via immunogold electron microscopy (EM) [66,108]. It was noted that O1-specifc antibodies bound equally well throughout the K2 capsule layer, recognizing LPS structures close to the bacterial surface (membrane proximal) as well as those that were further away (membrane distal). Anti-O antibodies were also capable of binding to outer membrane vesicles. Conversely, antibodies against the K-antigen primarily recognized membrane-distal epitopes located at the periphery of the capsule and resulted in capsular swelling, also known as the Quellung reaction.

#### 3.3.2. Capsular Masking of LPS

Although O-specific antibodies can bind the surface of intact *K. pneumoniae*, there are several examples where maximal opsonization occurred in the absence of a fully developed capsule. Rabbit antisera directed against the O-antigen showed more pronounced surface binding via whole-cell ELISA to K1, K10, K16, and K66 unencapsulated mutants than to the isogenic encapsulated strains [66]. This was also observed in mice vaccinated with O-antigen bioconjugates where O1-specific IgG bound significantly more to *K. pneumoniae* in the absence of K1 or K2 capsules. Furthermore, surface binding of anti-O antibodies in the bioconjugate antisera tended to be higher in *K. pneumoniae* strains that expressed lower quantities of K-antigen [86,87]. Reducing CPS synthesis through salicylate treatment can enhance exposure of subcapsular antigens and this was shown to increase reactivity of a murine anti-O1 LPS IgG3 mAb via ELISA across serotypes K1, K2, K10, K16, and K66 [109]. High-speed centrifugation can physically disrupt the capsule, which was sufficient to increase anti-O binding to an O1:K1 strain [51]. Other groups have corroborated these findings using similar assays and murine mAbs recognizing either the core or O-polysaccharide of O1 LPS [60,86,102,109,110,111].

Capsule-independent binding of O-specific antibodies has also been described. Two mouse IgG mAbs recognizing O1 O-antigen bound robustly to both a K2 encapsulated strain and an isogenic, unencapsulated mutant via flow cytometry [67]. More recent antibody discovery campaigns, employing phage display technology or probing antigen-specific B cell responses from humans, have identified O1 and O2 LPS-specific IgG mAbs with binding properties that are either impeded or unaffected by the presence of a capsule in flow cytometry or immunofluorescence assays [33,112].

The impact of *Klebsiella* spp. capsules on the effector functions of O-antigen-specific antibodies is a growing area of research. One report used flow cytometry to demonstrate that murine anti-O1 IgG1 and IgG3 mAbs were better able to agglutinate unencapsulated *K. pneumoniae* compared to its K2 encapsulated parent [67]. Another study comparing various K types (K1, K2, K10, K16, and K66) found that when capsule production was reduced via salicylate treatment, OPA with anti-O1 rabbit sera and human neutrophils was either diminished, equivalent, or enhanced compared to the untreated bacteria [109]. The K2 capsule was shown to inhibit the SBA of O1-specific antibodies against a respiratory isolate as well as a hypermucoid and mouse virulent strain of *K. pneumoniae* [86]. Finally, the quantity of CPS in a panel of 14 *K. pneumoniae* clinical isolates was inversely correlated with the level of complement-mediated bactericidal activity mediated by murine anti-O antibodies [87].

Collectively, the literature provides mixed evidence for the ability of *K. pneumoniae* capsules to block antibody access to subcapsular antigens. An important caveat is that thus far for *K. pneumoniae*, capsular masking of LPS has largely been observed in vitro, whereas a protective role in vivo for LPS-specific antibodies (either actively induced or administered passively) is documented across several animal studies with few exceptions. The apparent discrepancy may relate to differences in how *Klebsiella* spp. regulate CPS production during bloodstream infection versus culture conditions. Furthermore, capsule masking does not necessarily result in complete loss of anti-LPS antibody activity, and the phenomenon is not evident across all *K. pneumoniae* K-types and strains within a K-type. The nature of the capsule layer (i.e., length, sugar composition, conformation, and density) may therefore be critical in shaping LPS accessibility to antibodies (summarized in Figure 1) as was originally hypothesized by Williams and Tomás [50,51]; although, mechanistically, it is still unclear how this occurs in vivo.

### 3.4. Antibody Specificity—Accessibility and Protection

#### 3.4.1. Distance from the Outer Membrane

The O-antigens from *K*. *pneumoniae* serotypes O1 and O2 share a conserved backbone of galactose disaccharide repeating units referred to as ᴅ-galactan-I (gal-I). In O1 strains, gal-I can be capped at the nonreducing terminus with a polymer of antigenically distinct galactose disaccharides referred to as ᴅ-galactan-II (gal-II) (reviewed in [113]).

There are conflicting reports surrounding the importance of epitope distance from the *K. pneumoniae* bacterial surface on antibody-mediated protection. The membrane proximal lipid A-core region is a known target of protective antibodies in the context of bacteremia caused by O1:K2 *K*. *pneumoniae* [89,90,91,100]. Hsieh et al., however, reported that in response to a live-attenuated *K. pneumoniae* vaccine, protection against systemic O1:K2 challenge in mice was largely influenced by antibodies specific for the membrane-distal gal-II repeat units of O-antigen [77]. Pennini et al. supported this finding using immunofluorescence, demonstrating that the gal-II region of O1 LPS can shield the underlying gal-I antigen from antibody binding [33]. This study further reported that a gal-1-specific mAb afforded negligible to moderate protection against *K. pneumoniae* O1 pneumonia and bacteremia, respectively.

All of the major carbohydrate domains of O1 LPS are reportedly accessible to specific antibodies in vitro in whole-cell binding experiments with encapsulated *K. pneumoniae* [33,100,101,109,110,111,112]. One notable study compared a trio of anti-LPS mAbs (recognizing either the core oligosaccharide [IgG2a], the gal-I repeating units [IgG3], or the gal-II repeating units [IgG2b]) in whole-cell ELISAs, demonstrating that none could effectively opsonize O1:K2 *K. pneumoniae*, whereas partial binding was observed for the K7 and K21 serotypes [110]. This was associated with either a lack of bactericidal activity for the K2 capsules or heterogeneity in killing for the K7 and K21 capsules that was strain dependent. In the O1:K7 and O1:K21 serotypes, the anti-gal-II mAb showed the highest degree of binding and bactericidal activity. Anti-K mAbs were also tested and shown to mediate robust opsonophagocytic killing that was superior to the anti-O1 antibodies except for one O1:K21 strain. In the absence of a capsule, anti-gal-II mAbs elicited near maximal killing, but interestingly, the level of OPA with mAbs directed against the core and gal-I domains was strain dependent.

#### 3.4.2. Impact of O-Antigen Modifications on Antibody Accessibility

Carbapenem-resistant isolates of *K. pneumoniae* belonging to the ST258 clonal group are emerging as an important cause of hospital-related deaths globally. Many O1 and O2 ST258 isolates contain the *gmlABC* gene cluster, which modifies the gal-I repeating units with a terminal α-ᴅ-Gal*p* side group, creating an antigenically distinct galactose polymer referred to as ᴅ-galactan-III (gal-III) [55,114]. Gal-III type O-antigens from several encapsulated ST258 clinical isolates were surface accessible to a gal-III-specific mAb via flow cytometry [55].

The impact of the branched α-ᴅ-Gal*p* modification on antibody accessibility to other regions within the O-antigen is beginning to be explored. A computational molecular dynamics study suggested that the branched structure of gal-III O-antigen minimizes exposure of the underlying gal-I backbone to antibodies, whereas binding to the terminal gal-II region remains unaffected [115]. Supporting these simulations, plasmid expression of the *gmlABC* operon in gal-I^+^ O2 *K. pneumoniae* strains, which converts O-antigen into gal-III, can disrupt the interaction of a gal-I-specific mAb with LPS in Western blotting assays [55]. Another study, however, comparing *gmlABC*^+/-^ *K. pneumoniae* strains of varying sequence type observed comparable binding of gal-I and gal-II-specific mAbs to the bacterial surface via flow cytometry [112]. Some studies suggest that the degree of gal-I-specific antibody binding to LPS may be strain dependent [55,116,117].

#### 3.4.3. Cross-Reactive Antibody Responses to *K. pneumoniae* O-polysaccharide

Antibody cross-reactivity amongst the galactan-based *K. pneumoniae* O-antigens has been reported. Using an scFv phage display on whole *K. pneumoniae*, Berry et al. identified a human IgG1 mAb with specificity for both *K. pneumoniae* O1 and O2 LPS [112]. Therapeutic delivery of the anti-O1/O2 mAb to mice protected against lethal pneumonia induced by *K. pneumoniae* strains bearing either O1 LPS (clonal group ST25) or gal-III+ O2 LPS (clonal group ST258). Immunofluorescence microscopy revealed strong reactivity of this mAb with O1 *K. pneumoniae* (regardless of whether gal-III type O-antigen was present), and despite in vivo evidence to the contrary, weak binding to an O2 strain that was lost when gal-III was expressed. Opsonophagocytic killing activity was only observed in the absence of a capsule. Cross-reactivity is not always apparent. In a separate report, serum from rabbits immunized with a semisynthetic O1:CRM_197_ conjugate recognized O2 LPS from gal-I+ and gal-III+ *K. pneumoniae*, whereas serum IgG raised against a synthetic gal-III O2:CRM_197_ conjugate bound selectively to gal-III+ O2 LPS [81].

O1/O2 cross-protection studies have yielded conflicting results. A human anti-O1/O2 IgG1 mAb failed to protect against O1 pneumonia in mice but improved survival outcomes against O2 pneumonia and bacteremia, compared with monovalent antibodies given at similar doses [33]. An in vitro characterization of this mAb revealed intense binding to the surface of the O2 strain via immunofluorescence that was associated with robust opsonophagocytic killing; conversely, punctate binding was observed to the O1 strain accompanied by weak bactericidal activity. The anti-O1/O2 mAb effectively inhibited TLR4-MD2 signaling in vitro following O1 and O2 LPS stimulation. Hsieh et al. found that immunization of mice with a *K. pneumoniae* O1 Δ*magA* Δ*wbbY* mutant, which is acapsulated and lacks the distal gal-II repeats of O-antigen, failed to protect against an IP challenge with the parent O1:K2 strain [77]. Passive transfer of the O1 Δ*magA* Δ*wbbY* immune mouse sera was also less effective at clearing *K. pneumoniae* O1:K2 after challenge as compared to O1 Δ*magA* mouse antisera containing gal-II-specific antibodies. In contrast to this study, Clements et al. observed that O1 LPS-vaccinated mice experienced a significant delay in mortality following systemic challenge with an O2:K1 strain [67]. Key differences between these studies include the strain and K type of the *K. pneumoniae* used for challenge.

Antibody cross-reactivity has also been described for the *K. pneumoniae* O3 and O5 serotypes, which express O-antigens built of mannose-rich repeating units that can be terminated in methyl or methyl phosphate groups, respectively [113]. The O3 serotype is a collection of three sub-serotypes (O3, O3a, and O3b) that have repeating units of either tri, tetra, or pentasaccharide. O5 O-antigens have trimeric mannose repeating units and differ by the nature of their mannose linkages and anomeric configurations.

After immunizing mice with an O3a:K11 strain, Guachalla et al. uncovered two types of mAbs with broad specificity for O3 O-antigens using immunoblotting and flow cytometry: (1) antibodies with equivalent reactivity to LPS from sub-serotypes O3a and O3b and (2) antibodies that bound strongly to the immunizing strain LPS with relatively weak cross-reactivity to other O3 subtype LPSs [118]. Interestingly, despite their specificity for mannan O chains, none of the mAbs recognized O5 LPS. Cross-reactive antibodies to the mannose-based O-antigens have also been isolated from humans. Here, a panel of mAbs with varying degrees of cross-reactivity to O3a, O3b, and O5 LPS (as measured with ELISA, Western blot, and flow cytometry) was isolated from plasmablasts and memory B cells of healthy human donors [119]. Passive transfer of an O3a/O3b-specific mAb protected galactosamine sensitized mice against intravenous challenge with *K. pneumoniae* O3a and O3b strains. In vitro functional activity was also demonstrated for this mAb in the form of complement-dependent opsonophagocytic uptake and SBA to *K. pneumoniae* O3, O3a, and O3b serotypes.

## 4. Forging the Path to a Promising LPS-Based Vaccine Against *K. pneumoniae*

*K. pneumoniae* remains a major cause of morbidity and mortality worldwide, and there is an urgent need for countermeasures that operate independently of antibiotics. Vaccination could become an effective way to manage the spread of *Klebsiella* disease, and a variety of vaccine concepts targeting the core and O-antigen of *K. pneumoniae* LPS have been described. Important questions remain to be answered that could impact a candidate’s advancement through the development process and its prospects for licensure.

### 4.1. Breadth of Protection

Currently, there are four prevalent disease-causing serotypes (O1, O2, O3, and O5), and as discussed above, *Klebsiella* spp. can modify their O-antigen creating unique sub-serotypes. Which OPSs should be included in a *K. pneumoniae* vaccine to enable the broadest coverage globally? Shared epitopes reside within the galactan-based (O1/O2) or mannan-based (O3/O5) O-antigen families for *Klebsiella* spp.; however, our knowledge regarding this cross-reactivity stems largely from structural characterization with mAbs. The extent to which these highly specific antibodies reflect the polyclonal response to O-antigen, with diverse antibody specificities and effector functions, is unknown. More research is needed to establish the breadth of protection elicited via LPS-based vaccines against *Klebsiella* spp. This will inform optimal vaccine formulations and whether subtype-specific O-antigens are needed to ensure consistent efficacy over time. Additionally, broad spectrum strategies leveraging immunogenic and highly conserved subcapsular antigens, such as the core oligosaccharide, may create opportunities for serotype-independent immunity, including against strains that lack or have shorter OPS.

### 4.2. Capsular Masking of LPS

Hypermucoviscous strains of *K. pneumoniae* that express large quantities of CPS have raised concern over whether such capsules can mask the bacterial surface from immune recognition and whether an LPS-based Klebsiella vaccine would require an additional K-antigen component for optimal protection. The experimental evidence to date indicates that *Klebsiella* spp. capsules can disrupt LPS-specific antibody binding and functional activity but that this is not a universal phenomenon. Future studies should account for the potential impact of capsule masking under physiological conditions by evaluating protective efficacy in an animal challenge model using virulent strains with clinically relevant O- and K-antigen combinations where possible. Furthermore, given the large number of K-types among invasive isolates, molecular dynamics simulations may become a useful tool to model O-specific antibody binding in the context of diverse K-antigens, and to better understand why some K-types are more prone to capsule masking than others. Such computational methods have recently simulated how *S*. Typhimurium O-antigen sugar composition and outer membrane protein size work in concert to influence the formation of channels within LPS, thereby affecting antibody access to the bacterial surface [120]. Whether anti-LPS antibodies derived from vaccination will be able to bypass the capsule and protect against *K. pneumoniae* in humans is unknown. Phase 3 efficacy studies are required to ascertain how the capsule might impact the effectiveness of vaccination with *K. pneumoniae* LPS or its derivatives.

### 4.3. Combination Vaccines

While both LPS and CPS from *K. pneumoniae* have been well documented as protective antigens in animal models [121], little is known about how antibodies recognizing these targets function in the presence of one another. When encapsulated *K. pneumoniae* are placed in contact with K-specific antibodies in vitro, the capsule can swell in size (Quellung reaction) and undergo drastic morphological changes where the outer capsule layer becomes fibrous and less densely packed, with antibodies condensed at the capsule periphery, as observed via immunogold EM. This outer rim of bound antibody was hypothesized by Meno et al. to form an impermeable shell to macromolecules [108]. Promising K-antigen-based conjugate vaccines targeting the most common K-types associated with invasive *K. pneumoniae* infections, including K1 and K2, have been described [122,123]. Future research might address whether antibody-mediated capsular swelling of *K. pneumoniae* occurs under physiological conditions and to what extent this reaction precludes antibody binding to subcapsular targets. Furthermore, head-to-head studies comparing the performance of LPS-targeting *K. pneumoniae* vaccines to composite formulations containing both O and K antigens would be informative.

### 4.4. Correlates of Protection

There are no established correlates of protection against *Klebsiella* spp. Passive transfer studies in mice suggest that antibody responses against LPS are sufficient for protection against experimental *K. pneumoniae* infections. However, a deeper analysis of LPS-specific humoral immunity is lacking where epitope specificity, affinity, isotype, Fc glycosylation patterns, and functional activity are linked. An additional challenge is that the in vivo mechanisms of antibody-mediated protection against *K. pneumoniae* at different anatomical sites of infection are not clearly defined. For example, to prevent mortality from *K. pneumoniae* sepsis, protective antibodies will likely need to be operational within the circulation. However, hospital-acquired *K. pneumoniae* bloodstream infections are often preceded by colonization of the respiratory, gastrointestinal, or urinary tracts [124]. Within these sites, antibodies may act through distinct effector functions (e.g., IgA-mediated bacterial enchainment [125] or Fc-mucin cross-linking [126] to limit bacterial egress to normally sterile areas. Mucosal-associated antibodies may also reduce *K. pneumoniae* carriage and disrupt transmission. A comprehensive picture of immunity against *Klebsiella* spp. is needed to define robust correlates of protection which can then be applied as immunological endpoints to develop more effective vaccines.

### 4.5. Polymicrobial Infections and Symbiosis

Infections caused by CRKP can be complicated by co-colonization or co-infection with other carbapenem-resistant pathogens including *P. aeruginosa* and *A. baumannii*, which contributes to an increased healthcare burden through prolonged hospital stays, higher readmission rates, and patient mortality [127]. This may be related to symbiotic interactions between species. For example, co-culture of *K. pneumoniae* and *A. baumannii* can enhance the growth, biofilm formation, and virulence of both bacteria in a *Galleria mellonella* infection model relative to single-species cultures [128]. While bloodstream infections and urinary tract infections are often monomicrobial, it will be important to consider the potential impact that bacterial symbiosis can have on *K. pneumoniae* virulence and its effect on vaccine efficacy, particularly against bacterial pneumonia. Finally, disrupting polymicrobial populations through vaccination may also have an overall positive benefit. As an example, receipt of pneumococcal conjugate vaccines can confer protection against viral-associated pneumonia and lower respiratory tract infections, presumably through reductions in *S. pneumoniae* colonization [129,130]. Therefore, an efficacious *Klebsiella* spp. vaccine could have an indirect effect against pathogens that are unrelated.

## 5. Concluding Remarks

In conclusion, we have summarized the current understanding of LPS as a vaccine target for *K*. *pneumoniae* and the role of LPS-specific antibodies in protection against *K. pneumoniae* disease. Examination of the literature provides compelling evidence that *K. pneumoniae* LPS is accessible to specific antibodies that can mediate protective immunity against fatal experimental infection. A variety of vaccine technologies have been described where LPS is either specifically targeted or would be expected to play a significant role in protection. Overall, we believe that LPS-based vaccines will become an important component of the arsenal against *Klebsiella* spp. Continued efforts to address potential development challenges are needed to accelerate the evaluation and licensure of promising vaccine candidates and to maximize their impact.

## Figures and Tables

**Table 1:** LPS-targeting vaccines against *K*. *pneumoniae* in preclinical development.

Vaccine Type	Vaccine Components	Administration	Vaccine Serotype(s)	Immunogenicity	Efficacy	Reference
LPS	LPS	2 × IP (mice) 3 x (rabbits)	O1	Rabbit anti-LPS IgG-labeled O1 strains of various K-types (K2, K7, K8, K12, K19, K21, K22, K27, K34, K35, K42, K45, K55, K57, K62, K64, K66, K69, and K70) but failed to label serotypes K1, K10, and K16.	Protection in mice against IP challenge with a highly virulent K2 strain (4 log increase in LD50) and moderately virulent K21 and K61 strains (~1.5 log increase in LD50). Modest protection against a highly virulent K1 strain (~1.5 log increase in LD50).	[66]
		2 × IP (mice)	O1	Anti-O1 mAbs (IgG1 and IgG3) bound to and agglutinated O1:K2 in the absence of capsule.	Protection against IV challenge with both homologous (O1:K2) and heterologous (O2:K1) *Kp* when challenge occurred 14 days post-immunization (VE 100%) **. Efficacy waned 28 days post-immunization; however, a delay in mortality was seen.	[67]
		1 × IM or IU (mice)	O1	Unknown.	Reduced kidney burden following IU challenge with an O1:K2 strain.	[68]
	LPS-loaded liposomes	1 × IM (rats)	O1	LPS-specific B cells in the spleen.	Reduced lung burden following IT challenge with an O1:K2 strain.	[69]
	LPS encapsulated in alginate microparticles	1 × IN, IM, or IT (mice)	O1	All administration routes led to serum anti-LPS IgG responses.	Reduced lung burden after IT challenge with an O1:K2 strain. IT vaccination resulted in complete elimination of challenge strain.	[70]
Whole cells	Formalin-inactivated *Kp*	4 × IP (mice)	O?:K1	Unknown.	Protection against lethal IP and aerosol challenge with vaccine strain (VE 80–90% and 50%, respectively)	[71]
	Heat-killed *Kp*	2 × IN (mice)	O1:K2	Serum IgG reacted with K2 (67% of strains) but not K1 or K16 serotypes. Th17 response observed in the lungs.	Reduced bacterial burden in lungs and spleen after IT challenge with vaccine strain. Cross-protection against serotypes K1 and K16 (O-undetermined) seen in the lungs.	[72]
		3 × ID (mice)	Unknown	Anti-*Kp* IgG detected in sera that demonstrated SBA activity.	Protection against lethal IP challenge with vaccine strain (VE 50–100%).	[73]
	*Kp* Δ*tonB* (deficient in iron uptake)	1 × IP (mice)	O1:K1	Serum IgG detected against extracellular polysaccharide antigens.	Protection against lethal challenge with wild-type strain (VE 100%).	[74]
	*Kp* Δ*murl* (glutamate auxotroph)	2 × IP (mice)	O?:K52	Serum IgG and IgM detected against parental strain. IgG cross-reacted with other K-types (K1, K2, K17, and K24) belonging to different sequence types. Vaccination triggered splenic IL-17 and IFNγ production.	Protection against lethal IP challenge with the parental strain (VE 100%). Efficacy observed against serotype K2 (delay in time to death) and K17 (VE 67%) as well as a strain with an undetermined K-type (VE 100%).	[75]
	*Kp* Δ*kbvR* (biofilm and virulence deficiency)	2 × SC (mice)	O1:K1	Serum IgG recognized the wild-type strain. Antisera displayed OPA and SBA activity against O1:K1 and O?:K3 strains.	Protection against lethal IP challenge with parental strain via active and passive immunization (VE 88% and 40%, respectively). Reduced bacterial burden in blood, peritoneum, liver, spleen, and lung.	[76]
	*Kp* Δ*magA* (capsule deficient mutant)	3 × IP (mice)	O1	Serum anti-LPS IgG confirmed in pooled mouse sera.	Protection against lethal IV challenge with parental strain (O1:K2) (VE 70%). No protection against O2:K2 challenge. Passive transfer of immune mouse sera reduced bacterial burden in spleen and liver.	[77]
Conventional conjugates	COPS:TT	1 × IM (mice)	O1	Conjugate-induced sera agglutinated the challenge strain. Displayed OPA activity with alveolar macrophages.	Decreased lung burden after pulmonary challenge with an O1:K2 strain.	[78]
	COPS:FlaA COPS:FlaB	3 × IM (mice) 4 × IM (rabbits)	O1, O2, O3, O5	Serum anti-COPS IgG detectable in vaccinated mice and rabbits. O1 conjugate antisera from mice recognized various O1 strains from different K-types (K1, K3, K12, K22, K23, K24, K28, K38, and K62). Quadrivalent rabbit antisera induced OPA activity against O1:K2 and O3:K? isolates.	Passive protection after IV challenge with an O1:K2 strain (VE 78%). Reduced bacterial burden in blood, liver, and spleen for O1 and O3 strains.	[79]
	COPS:BSA	2 × IP (mice)	O1	Unknown	Protection after IP challenge with the O1 vaccine strain (VE 100%).	[80]
Semisynthetic conjugates	Oligosaccharides coupled to CRM_197_	3 × SC (mice & rabbits)	O1, O2a, O2ac, gal-III+ O2	High antibody titers against synthetic glycans and corresponding LPSs. O1-specific rabbit antisera cross-reacted with all O2 LPS subtypes. Gal-III-specific rabbit antisera only reacted with homologous LPS.	Unknown.	[81]
		3 × SC (mice & rabbits)	O3, O5	High antibody titers against synthetic glycans and corresponding LPSs.	Unknown.	[82]
Biosynthetic conjugates	OPS:CTB	3 × IP (mice)	O2	Multiple serum IgG subclasses observed following immunization.	Protection after IP challenge with O2 strain (VE 71% with adjuvant formulation)	[83]
		3 × SC (mice)	O2	Serum anti-O2 IgG titers appear after 2nd immunization. Multiple IgG subclasses observed.	Protection after IP and IT challenge with an O2 strain (VE 100% and 60%, respectively). Reduced bacterial burden in blood, liver, spleen, and lungs.	[84]
	OPS:AP205	3 × SC (mice)	O1, O2	Elevated serum IgG against O1 LPS after 3rd immunization.	Protection after IP challenge with an O1 strain (VE 100%) and an O2 strain (VE 50%). Efficacy against the O2 strain increased to 70% after doubling the amount of polysaccharide.	[85]
	OPS:EPA	3 × SC (mice)	O1, gal-III+ O1, O2a, gal-III+ O2, O3, O3b, O5	Serum anti-O IgG in response to monovalent and heptavalent formulations. Cross-reactivity noted for monovalent IgG amongst and between the O1/O2 subtypes and within the O3 subtypes. Heptavalent antisera demonstrated variable bacterial surface reactivity and SBA activity. Increasing quantities of CPS correlated with decreasing capacity of antisera to bind and mediate SBA.	No protection against IT or IP challenge with O1:K2 strains following O1 OPS:EPA immunization. Efficacy was not determined for the heptavalent formulation.	[86,87]
Multiple antigen presentation system (MAPS)	12 polysaccharides (4 *Kp* COPS and 8 *Pa* COPS) and 3 proteins (*Kp* MrkA, *Pa* flagellin B, and *Pa* PcrV) linked to K19 CPS from *Klebsiella. oxytoca*.	2 × IP (rabbits)	O1, O2, O3, O5	IgG seroconversion to all 4 COPS antigens observed after 2 doses in rabbits. Vaccine-induced antibodies mediated OPA of O1 and O3 strains. MAPS antisera bound to and agglutinated intact *Kp* across several non-vaccine O-types.	Passively protected mice against IP challenge with an O1:K2 strain (VE 40%). Reduced bacterial burden in blood and spleen following IV challenge.	[88]
Noncovalent complexes	Delipidated inner core OS from *E. coli* J5 (Rc) admixed with porB from *Neisseria meningitidis*	2–3 × SC (rat)	N/A	Serum IgG responses of multiple subclasses induced in rats following subcutaneous immunization.	Protection after IP challenge with an O1:K2 strain (VE 70%). Reduced bacterial burden in blood, liver, and spleen. Reduced circulating endotoxin.	[89]
		1 × IM 2 × IP 2 × SC (mice)	N/A	Elevated serum IgG responses following IM and IP immunization. OPA activity demonstrated in post-vaccination sera.	Protection after subeschar challenge with an O1:K2 stain (VE 30%).	[90]
		3 × IN or IP (mice)	N/A	Multiple serum and bronchiolar lavage IgG subclasses observed after immunization. OPA activity demonstrated in post-vaccination sera.	Protection after IT challenge with an O1:K2 strain (VE 67%). Reduced bacterial burden in the blood, spleen, liver, and lungs.	[91]
Outer membrane vesicles (OMVs)	*Kp* extracellular vesicles (EVs)	3 × IP (mice)	Unknown	EV-specific IgG production and a strong Th1, IFN-γ-producing T cell response.	Protection against IP challenge with O?:K1 (VE 100% with 1 μg EVs). Protection following adoptive transfer of sera or splenocytes from EV-immunized mice (VE 70% and 100%, respectively)	[92]
	BSA nanoparticle (BN) reinforced *Kp* OMVs	3 × SC (mice)	Unknown	Serum IgG specific to an ST11 carbapenem-resistant *K. pneumoniae* (CRKP).	Protection after SC challenge with CRKP (VE 100%). Protection following adoptive transfer of sera and splenocytes from BN-OMV-immunized mice (VE 67% and 100%, respectively).	[93]

Abbreviations: *Kp*—*Klebsiella pneumoniae*; *Pa*—*Pseudomonas aeruginosa*; LPS—lipopolysaccharide; IM—intramuscular; IP—intraperitoneal; IU—intraurethral; IV—intravenous; IT—intratracheal; SC—subcutaneous; VE—vaccine efficacy; mAb—monoclonal antibody; OPA—opsonophagocytic antibody; SBA—serum bactericidal antibody; N/A—not applicable. ** Vaccine efficacy (VE) refers to the relative survival in control and vaccinated groups and is defined as ((control attack rate − vaccinated attack rate)/control attack rate) × 100. It is expressed as a percentage.

## Data Availability

No new data were created or analyzed in this study. Data sharing is not applicable to this article.
